# Evaluation of single nucleotide polymorphism in interleukin 22 (IL-22) gene and its association with chronic hepatitis B infection 

**Published:** 2019

**Authors:** Paniza Asadi, Seyed Reza Mohebbi, Seyed Masoud Hosseini, Mohammad Reza Zali

**Affiliations:** 1 *Basic and Molecular Epidemiology of Gastrointestinal Disorders Research Center, Research Institute for Gastroenterology and Liver Diseases, Shahid Beheshti University of Medical Sciences, Tehran, Iran*; 2 *Department of Microbiology and Microbial Biotechnology, Faculty of Life Sciences and Biotechnology, Shahid Beheshti University, Tehran, Iran*; 3 *Research Center for Gastroenterology and Liver Diseases, Research Institute for Gastroenterology and Liver Diseases, Shahid Beheshti University of Medical Sciences, Tehran, Iran *

**Keywords:** Interleukin 22, Single nucleotide polymorphism, Hepatitis B virus, Immune response, Inflammation

## Abstract

**Aim:**

This study aimed to evaluate rs1179251 single nucleotide polymorphism in the IL-22 gene as a host factor and its effect on chronic hepatitis B infection.

**Background:**

Interleukin 22 (IL-22) belongs to a group of recently discovered cytokines, and it is produced and secreted by innate lymphoid cells (ILCs) and T helper 22 (Th22) cells. This cytokine plays dual roles as pro-inflammatory and anti-inflammatory effects in various conditions and different tissues of the body.

**Methods:**

This study was performed based on a case-control format to assess IL-22 rs1179251 single nucleotide polymorphism genotypic and allelic frequencies among 227 hepatitis B chronic patients and 227 healthy controls. The polymerase chain reaction and restriction fragment length polymorphism techniques were employed to determine the polymorphism’s genotypes.

**Results:**

Genotypes Frequencies in patients’ group were determined CC 59.91%, CG 37.89%, and GG 2.20% respectively in comparison to CC 63.44%, CG 31.72% and GG 4.85% in control group. The findings revealed that there was no statistically significant difference in the genotypes (P=0.156) frequencies of IL-22 gene polymorphism (rs1179251) between patients and control groups.

**Conclusion:**

No association was found between rs1179251 single nucleotide polymorphism in IL-22 gene and chronic hepatitis B infection. So, in spite of the importance of IL-22 gene in immune responses, the studied polymorphism does not serve a decisive role in susceptibility to hepatitis B virus chronic infection.

## Introduction

 Chronic hepatitis B disease is known as one of the leading world health problems, and infection with hepatitis B virus (HBV) can lead to the disease. Approximately 400 million people are infected with HBV around the world (1, 2).

In Iran, several epidemiological, serological, and molecular surveys have been conducted on HBV, and their results revealed the importance and risks of HBV infection on public health (3-7).

Hepatitis B virus chronic infection can develop severe liver complications, including liver cirrhosis and hepatocellular carcinoma (HCC). However, HBV infected patients may face different levels of disease depending on critical factors such as virus characteristics and also the functional capability of the immune response which the latter is directly associated with the host genetic background. Cytokines are important protein molecules and are involved in immune responses. They may be produced and secreted in reaction to stimulants from various host cells and act as signal transfer between different cells and tissues. Cytokines can induce inflammation and anti-tumor functions and also may begin and progress innate and adaptive immune responses (8, 9).

Interleukins are a broad group of cytokines which play important regulatory roles in the immune system through distinct approaches. IL-22 is a cytokine member of IL-10 superfamily that can attach to its receptors IL10R2 and IL22R1 on the surface of the relevant cells and mediates cellular inflammatory responses (10). 

IL-22 is produced by activated dendritic and T cells and can stimulate innate immune responses against pathogens. IL-22 Biological functions initiate with the attachment of the ligand to the specific receptors, and consequently, regulation and induction of responses begin. IL-22 target cells are mainly hepatocytes, keratinocytes, lung, and gut cells. It seems that production and secretion of cytokine might be associated with genetic polymorphisms and can provoke a possible increase or decrease in the intensity of immune responses and consequently, in disease progression and outcome (11).

Recent studies revealed a probable link between IL-22 and liver viral infections. IL-22 plays two leading roles (pro-inflammatory and protective) in the body. This cytokine protects hepatocytes by induction of liver cells growth and regeneration. It can also increase chemokine production and promote the expression of antimicrobial proteins required for pathogens elimination (12-14). IL-22 receptors are generally expressed and located on specific cells such as hepatocytes in liver tissue, and IL-22 signaling can lead to pro-inflammatory proteins, activation of anti-apoptosis processes (15). 

This study aimed to evaluate rs1179251 single nucleotide polymorphism in the IL-22 gene as a host factor and its effect on chronic hepatitis B infection. 

## Methods

**Study population**

 The present survey was performed on four hundred and fifty-four subjects, including two hundred and twenty-seven HBV chronically infected patients and the same number of healthy individuals as a case-control cross-sectional survey. The patients and control individuals were referred to Tehran Taleghani Hospital during 2014-2016. Inclusion criteria for the present study were the previous diagnosis of chronic HBV infection for the case group. For the control group, lacking any history of hepatitis and infectious liver diseases was the inclusion criteria. Blood samples were collected from study subjects, and confirmatory serological tests were done. Competitive Enzyme ImmunoAssay (ELISA) for the determination of antibodies to Hepatitis B core Antigen in the sera was utilized (Diapro Diagnostics, Italy). Also, the third-generation Enzyme Immunoassay for the determination of Hepatitis B surface Antigen or HBsAg in the serum samples was performed (Diapro Diagnostics, Italy). 

Informed consent was taken from each one of the individuals, and the study protocols have been assessed and authorized by the ethical committee of the Research Institute for Gastroenterology and Liver Diseases. 

**Sample preparation and genotyping**

**Figure 1:**
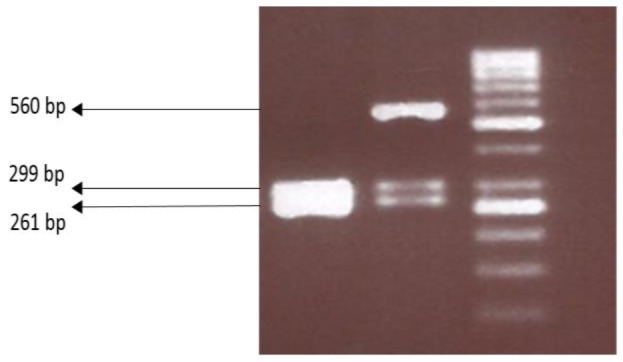
Digestion patterns of IL-22 gene SNP rs1179251 (AlwNI enzyme). The left column indicates the homozygote (CC) pattern, the middle one shows heterozygote (CG), and the right column is DNA size marker 50 bp

Genomic DNA of studied subjects was extracted applying salting out standard method. Polymerase chain reaction (PCR) and restriction fragment length polymorphism (RFLP) technique were utilized to verify genotypes. A pair of primers was designed, to amplify a region containing rs1179251 single nucleotide polymorphism, using Gene Runner software (Version.4.0.9.68) and checked by BLAST (Basic local alignment search tool, National center for biotechnology information, USA) (table 1). The following protocol was used to amplify the aforementioned region; 100 ng genomic DNA was added as the template to an end volume of 25 µL master mix of 1.25 U of Taq polymerase (YTA, Iran) and 1x buffer (MgCl2 plus, final concentration 1.5 mM), 1.5 µL dNTP (10mM) and one µL of each primer (10 pmol). The PCR was operated on automated thermocycler (Eppendorf, Germany) according to the following program; 94 °C for 5 min for first denaturation, then 35 cycles of 94 °C for 30 seconds, primers annealing step 60°C for 30 sec, 72 °C for 45 sec and a final extension step of 72 °C for 7 min. The PCR product then digested by AlwNI restriction enzyme (incubated at 37° C for 18 hours) and the ultimate product was run on 3% agarose gel and visualized under UV light (figure 1). To confirm the genotyping results, 10 percent of samples randomly selected and were re-genotyped using direct sequencing method (Macrogen, South Korea). 

**Table 1:** Properties of primers for amplification and enzyme for RFLP

Genotypes fragment sizes (bp)	Restriction enzyme and the recognition site	Annealing temperature	GC percent	Sequence	Primer name
GG: 547 GC: 547, 298, 249 CC:298, 249	AlwN I CAGNNN^CTG	60.0 °C	47%	5’- CAGAAATTAGCCCTATATGC -3’	IL-22-251-forward
			42%	5’- GAAAAAGGTAGGACTGATAAC -3’	IL-22-251-reverse

**Statistical methods**

 The results were analyzed by SPSS biostatistics version 20 (IBM, USA). Chi-square test, non-parametric Mann-Whitney test were used to evaluate the results. Variables differences with P values below 0.05 were considered as statistically significant.

## Results

Gender distribution of the studied subjects was as follows; in the HBV chronically infected patients 117 patients were male (51.54%), and 110 were female (48.46%), on the other hand, in the control group 100 individuals were male (44.05%) and 127 were female (55.95%). The mean age of patients was 39.54, and controls were 39.06 years (Table 2). There was not any statistically significant difference between case and control groups’ gender and age. 

**Table 2:** Age and gender distribution of the studied patients and controls

variables	Total studied subjects (n=454)	Patients (n=227)	Controls (n=227)	P value
Age *	39.30+13.41	39.54+13.55	39.06+13.29	0.851
Gender	0.110			
Male	217(47.8%)	117(51.5%)	100(44.1%)	
Female	237(52.2%)	110(48.5%)	127(55.9%)	

*years, Mean+SD

**Table 3:** Comparison of genotypes frequencies in IL-22 rs1179251 position between HBV chronically infected patients and healthy controls

P value	Controls	Patients	Rs1179251 Genotypes
0.156	144 (63.4%)	136 (59.9%)	CC
	72 (31.7%)	86 (37.9%)	CG
	5 (4.9%)	11 (2.2%)	GG

**Table 4:** Comparison of allele frequencies in IL-22 rs1179251 SNP among patients and control groups

P value	Controls	Patients	Rs1179251 allele
0.935	360 (55.8%)	359 (44.2%)	C
	94 (45.2%)	95 (54.8%)	G

The mean level of serum Alanine Aminotransferase (ALT) for patients was 42.78+38.29 IU/L and 22.84+12.65 IU/L for controls. 

Analyzing the genotyping results of IL-22 gene rs1179251 single nucleotide polymorphism revealed the distribution of CC (136 patients, 59.91%), CG (86, 37.89%) and GG (11, 2.20%) in the patients’ group and CC (144 individuals, 63.44%), CG (72, 31.72%) and GG (5, 4.85%) in the controls group. Based on the genotyping result, there is no statistically significant difference between case and control groups for IL-22 rs1179251 SNP. Genotypes and allele frequencies were presented in Tables 3 and 4.

## Discussion

Cytokines play principal roles in defense against viral infections. They can perform these roles directly by viral replication inhibition mechanisms or through indirect effects on modulating pattern of host immune responses. Host genetic background is a crucial factor which regulates cytokine responses, and this may link with inflammation, viral clearance, or disease progression. The association between single nucleotide polymorphisms and various clinical diseases such as colorectal cancer, gastric cancer, and autoimmune diseases or infectious diseases including HBV and HCV infections have been studied and proved in several different surveys (16-21). 

Interleukin 22 plays several significant roles in different human organs, especially in the liver. IL-22 may probably perform a protective or even therapeutic functions against HBV and HCV infections. At present, studies focus on the main role of IL-22 in the liver, which is protection against liver damage in chronic diseases and hepatocellular carcinoma. However, this cytokine shows both protective and also inflammatory functions and effects in various organs (22). 

The IL-22 protective role acts through production induction and increase of proteins responsible for hepatocytes proliferation, then protect and support liver tissue (23, 24). This process has been observed in chronic liver diseases such as the fatty liver. IL-22 can lead to higher production of anti-apoptotic proteins through the STAT3 pathway. 

Previous studies on IL-22 polymorphisms showed different results. Zenewic et al. reported that NK and T cells IL-22 can protect mice from inflammatory bowel disease at in vivo and invitro conditions (25). Another study revealed that IL-22 could protect enteric mucous from inflammation and heal intestine epithelial cells injuries through its anti-apoptotic properties (26). In 2008, Zhang and colleagues reported that IL-22 could increase survival of cancerous cells and their resistance to chemotherapy through the induction of antiapoptotic mediators. On the other hand, in another survey, it is claimed that IL-22 can lead to cell cycle arrest in mouse mammary cells. In 2010, a significant association between IL-22 polymorphism (rs1179251) and colorectal cancer had been reported (10-12, 27, 28). 

In 2018, Zhang et al. analyzed the association of four IL-22 gene polymorphisms (rs2227485, rs1179251, rs1179246, and rs1182844) and the risk of cancer in a recent meta-analysis. The results revealed the importance of rs1179251 polymorphism as a probable risk factor for cancer (29). 

More recent studies on IL-22 single nucleotide polymorphisms showed the importance of these variations in different diseases. Wang et al. analyzed the association of three IL-22 SNPs, including rs2227485, rs2227513, and rs2227491 with systemic lupus erythematosus (SLE) in a Chinese population (30). They found that individuals with rs222751 AG genotype showed lower serum levels of IL-22 in comparison with AA genotype. This study revealed an rs2227513 polymorphism might contribute to SLE susceptibility, possibly through reducing the expression of IL-22. 

In 2017, another recent study on infectious disease (cerebral malaria, CM) that induced by Plasmodium falciparum assessed 47 SNPs of IL-22 and IL-22RA2 in children from Mali and Nigeria and revealed the involvement of IL-22 SNPs in the pathogenesis of CM (31). The authors found a significant difference in rs1012356, rs2227476, and rs2227473 between children with CM and healthy controls. They claimed that SNP rs2227473 is placed in a vital production regulator position of IL-22. It seems that Individuals with T allele of rs2227473 express higher levels of IL-22 than those without this allele. 

Regarding HBV infection, the roles of IL-22 are not determined, and there are controversial results, and the possibility of IL-22 dual function should be considered (32-34). In patients infected with chronic HBV or HCV infections and in mice models studies, it has been reported that IL-22 production boosts in liver tissue (35, 36). Besides, serum levels of IL-22 HBV infected patients and number of Th17 (as a source of IL-22 production) are increased (37). Nevertheless, IL-22 direct antiviral effect is utterly insignificant, and it cannot promote classic IFN stimulated antiviral pathways and mediators (38). Feng et al. proposed that IL-22 may perform as proinflammatory function in the course of acute infection to clear the virus from host body but latter during chronic HBV disease, it possibly protects liver tissue (39). 

In conclusion, IL-22 is a significant cytokine product of Th17 and serves an essential function in inflammatory and liver diseases. It was expected to find a probable link between rs1179251 polymorphism and chronic hepatitis B virus infection. The present study is the first survey on the potential association between the SNP and HBV chronicity in a studied Iranian population. However, the results show that there is no association and rs1179251 cannot consider as an effective and important factor in host susceptibility to chronic HBV infection.
